# Food and Nutrition Insecurity in Selected Rural Communities of KwaZulu-Natal, South Africa—Linking Human Nutrition and Agriculture

**DOI:** 10.3390/ijerph14010017

**Published:** 2016-12-27

**Authors:** Laurencia Govender, Kirthee Pillay, Muthulisi Siwela, Albert Modi, Tafadzwanashe Mabhaudhi

**Affiliations:** 1 Dietetics and Human Nutrition, School of Agricultural, Earth and Environmental Sciences, University of KwaZulu-Natal, Private Bag X01, Scottsville 3209, Pietermaritzburg 3201, South Africa; govender.laurencia@gmail.com (L.G.); PillayK@ukzn.ac.za (K.P.); Siwelam@ukzn.ac.za (M.S.); 2 Crop Science, School of Agricultural, Earth and Environmental Sciences, University of KwaZulu-Natal, Private Bag X01, Scottsville 3209, Pietermaritzburg 3201, South Africa; modiat@ukzn.ac.za

**Keywords:** agriculture, balanced diets, food and nutrition security, human health, over- and under-nutrition

## Abstract

Lack of access to nutritious and balanced diets remains a major impediment to the health and well-being of people living in rural areas. The study utilizes a qualitative systematic approach to conduct an environmental scan and review of scientific literature of studies conducted in South Africa, specifically KwaZulu-Natal (KZN). Availability and access to nutritious, diverse and balanced diets were identified as key constraints for achieving food and nutrition security as well as for human health and well-being. This has led to both under- and over-nutrition, with the former, in particular stunting, affecting children under 5 years. A high incidence of over-nutrition, both overweight and obesity, was observed among black African females. In South Africa, poor people rely mostly on social grants and cannot afford a balanced diet. Under these circumstances, agriculture could be used to increase availability and access to diverse and nutritious foods for the attainment of a balanced diet. The wider use of traditional vegetable crops and pulses could improve availability and access to healthy and locally available alternatives. The promotion of household and community food gardens, and the use of nutrient dense crops with low levels of water use, i.e., high nutritional water productivity, offers prospects for addressing malnutrition in poor rural areas.

## 1. Introduction

Lack of balanced diets, manifested as under- and over-nutrition, is a major concern in South Africa [1] and other developing parts of the world. This has affected human health and well-being as observed by the increased risk of chronic lifestyle diseases such as obesity, hypertension, coronary heart disease and hyperlipidaemia [1,2,3,4,5]. There are several underlying causes of malnutrition in South Africa which are largely inter-linked, with poverty being the leading cause [6]. Poverty can be defined as the lack of or limited access to basic necessities, such as safe clean water, health care, shelter, sanitation, nutritious food and basic education due to economic constraints [7,8]. An estimated 47%–53% of black South Africans are affected by poverty, with the largest proportion of poor households being found in KwaZulu-Natal (KZN) [9]. Such poor households are often at a high risk of malnutrition as they cannot afford a balanced diet [10]. Several strategies such as biofortification and promotion of underutilised crops that are nutrient-dense have been proposed to improve dietary diversity and balanced diets [11,12] as part of attempts to improve the food and nutrition security of poor rural households.

Food and nutrition insecurity can be defined as the inability to access adequate quantities of nutritious foods required for optimal growth and development. There is a direct relationship between food and nutrition insecurity and poverty [13]. In South Africa, at the national level, food insecurity affects one in five households [14], many of which are located in rural communities. Most rural inhabitants consume diets that have very limited variety and are typically inadequate in fruits and vegetables [15]. In developing countries such as South Africa, not only is macronutrient deficiency a common problem, but also micronutrient deficiencies [16]. These include iron, iodine, zinc and vitamin A [6,17].

Various national studies have been conducted to assess the nutritional status of South Africans. The South African Vitamin A Consultative Group (SAVACG) conducted a survey in 1994 on children aged 0–71 months [18] while the National Food Consumption Survey (NFCS, 1999) surveyed children aged 1–9 years of age [19]. The National Food Consumption Survey-Fortification Baseline [14] was conducted on children aged 1–9 years and women of child bearing age and the most recent study, the South African National Health and Nutrition Examination Survey (SANHANES-1) was conducted in 2012 [20]. These studies have attempted to give an overall view of the nutritional status of the South African population. These studies also attempted to draw conclusions on the nutritional status of South Africans living in the different provinces. The sampling method used when conducting the national studies was disproportionate stratification by province. This means that only a certain fraction of the province was sampled. Therefore, the data obtained from these studies are arguably not a true reflection of the nutritional status of different population groups found in specific localities within the province, including district and sub-district levels [18,20]. This makes it challenging to come up with relevant, effective and sustainable intervention strategies to improve food and nutrition, and overall health status, of these communities.

The aim of the current review was to determine the nutritional status of people living in KZN, South Africa, especially poor rural people. This was achieved by evaluating available data and information on nutritional status of population groups residing in KZN. The focus on poor rural communities was derived from the fact that most of the national studies indicated that the poor rural population is the worst affected and most vulnerable to both under- and over-nutrition [14,19,20].

### 1.1. Overview of the KwaZulu-Natal Population

#### 1.1.1. Political Demarcations

KwaZulu-Natal is one of nine provinces located in South Africa [21]. This province covers an area of 92,100 km^2^ and shares borders with three other provinces, namely Eastern Cape, Mpumalanga and the Free State [22]. Pietermaritzburg is the capital city of the province and Durban is the largest city [22]. Within KZN, there is one metropolitan area (eThekwini Metropolitan), 10 districts and 50 municipalities. The districts and their local municipalities that are in the KZN province are shown in Table 1.

#### 1.1.2. Population Statistics and Distribution

KwaZulu-Natal has the second highest total population in SA [23] with an estimated population size of 10,919,100 people. This makes up 19.9% of the total population of SA [21]. KwaZulu-Natal comprises different population groups, namely, Africans (86.9%), coloured (1.3%), Indian (7.5%) and white (4%). The 2011 national census indicated that the majority of the population living in KZN were of black African ethnicity. From 1996 to 2011, a significant increase was noted in the black African population in South Africa, whilst a decrease was noted in the coloured, Indian and white populations [24]. In KZN, there was a decrease in the number of persons living in informal (8.3%) and traditional (19%) dwellings and an increase in persons residing in formal dwellings (71.6%) [24]. In 2001, it was noted that 46% and 54% of the KZN population lived in urban and rural areas, respectively [25]. On average, eight people resided in a single household in KZN [26,27]. The language spoken by the majority of the KZN population is isiZulu (80.9%) [28]. It was further noted that the majority of the population living in KZN were between the ages of 15–64 years. There were more females (52.5%) than males (47.5%) residing in KZN [24].

#### 1.1.3. Socio-Economic Status

KwaZulu-Natal comprises a heterogeneous population from different socio-economic backgrounds. In 2004, KZN had the third highest percentage of female-headed households (42.9%) [24]. Within the province, eThekwini (31.4%) had the highest percentage of female-headed households, followed by uMgungundlovu (10.6%) and uThungulu (8.8%). The lowest percentage of female-headed households was recorded in the Harry Gwala district (4.1%) [24]. The increase in the number of female-headed households may be attributed to the migration of males for employment [24]. Many of the South African population are unemployed and rely solely on grants for income [26,27].

Studies have shown that between 80%–81% of people residing in the uMkhanyakude and Zululand Districts in KZN receive child support grants, 16% received a salary and between 18%–27% relied on a partner or husband for financial support [29]. Another study showed that 30.2% of the participants were dependent on child support grants [30]. Although the 2011 census indicated that the average annual income earned in KZN was R 83,050 (USD 5869) and the unemployment rate dropped from 39.4% to 33% from 1996–2011 [24], many people still rely on grants for financial support. According to the budget speech of 2016, the following amounts were allocated for the various grants; child support grants (R 350), pension, disability and care dependency grants (R 1500 and will increase to R 1510 in October 2016) and foster grant (R 890) [31]. Majority of money received from grants is primarily used to purchase foods [32]. With the recent rise in food costs, it has become a challenge for poor people to access nutrient dense foods. As of June 2016, the cost of a food basket had increased to R 609.52 [33]. A possible strategy to improve dietary intake could be the promotion of food gardens at a household or community level in rural areas where there is access to land. This would assist in dietary diversity; improving food security as well as reducing income spent purchasing food [34,35].

In comparison to the 1999 NFCS study conducted in South Africa, the NFCS-FB 2005 study indicated a decline in food secure persons (19.8%), a 0.2% decrease in persons experiencing hunger and a 4.9% increase in persons at risk of hunger [14,19]. This highlighted increasing food insecurity in South Africa. Between 2005 and 2007, KZN was one of the provinces with the highest poverty rates, affecting 73.5% of children. The high incidence of poverty affecting children is of concern as this highlights risk of food and nutrition insecurity within this vulnerable group. Further, it was noted that 47% of people living in KZN survived on an income that was below the poverty line [24,29]. This was consistent with the huge proportion of persons who rely on government grants as their primary source of income. A more recent study conducted in 2012 indicated that persons that were at risk of hunger increased to 28.3%, however, food secure persons increased to 45.6% while persons experiencing hunger declined to 26% [20].

Although some improvement was observed in the percentage of food secure persons and those who experienced hunger, recent statistics have shown that on a national level, 58% of the rural South African population resides in areas that are economically unstable [28]. These persons have limited access to nutrient dense foods [16,28]. Unemployment rates and low levels of income have an effect on the nutritional status of persons residing in KZN. These contributing factors result in the consumption of diets that are high in carbohydrates, low in animal sources and lack variety, which results in micronutrient deficiencies [36,37]. Under these conditions, agriculture may be the suitable vehicle for addressing gaps in nutrition and incomes of poor rural people.

Several factors have been identified as being responsible for malnutrition. These include poverty, food and nutrition insecurity, inadequate infrastructure, limited access to healthcare facilities, low levels of education and inadequate food intake [6,13,38,39]. Poor rural people are the most affected by poverty and food and nutrition insecurity. This directly affects the nutritional status of this group.

## 2. Methodology

A qualitative systematic approach was adopted for the current review. Several databases which included; Google, Google Scholar, UNICEF website, WHO website, DoH website, Science Direct, Cochrane library and Pub Med, were used to search for key references. The key words that were used included; “nutritional status in KwaZulu-Natal”, “undernutrition”, “over-nutrition, obesity”, “malnutrition”, “food insecurity”, “nutrition insecurity”, “micronutrient deficiencies”, “KwaZulu-Natal”, “indigenous vegetables”, “wild vegetables”, “nutritional interventions”, “dietary diversity” and “link of water and nutrition”. There were over 3000 hits for the above keywords. However, the search was further filtered to include only those studies conducted in South Africa and KZN specifically. For the purpose of the literature review, only scientific articles, books and official websites were selected. Where international literature was cited, it was used to support local literature.

## 3. Results and Discussion

### 3.1. Dietary Intake, Balanced Diets, Human Health and Well-Being in KwaZulu-Natal

#### 3.1.1. Undernutrition

Undernutrition is regarded as a serious problem as it leads to poor quality of life due to the loss of body cell mass. It is associated with various health issues such as anaemia, hepatic mass losses, infection, emphysema, gastrointestinal (GI) tract atrophy and intestinal bacterial overgrowth [40]. Undernutrition is common in children. There are a number of indicators used to classify undernutrition in children using the WHO classification. Table 2 provides a detailed classification of malnutrition using anthropometric indicators. These indicators assist in determining if the child is underweight, stunted or wasted. These indicators were formulated using the WHO guidelines and are explained under the table [41,42].

##### Stunting

Stunting is a chronic form of malnutrition [45] and is defined as a height for age below the minus two standard deviation (SD) [46]. Stunting is caused either by inadequate food intake, or consumption of foods that lack adequate nutrients for an extended period of time [47]. It is a serious health problem as it can lead to reduced mental development, poor performance in school and increased risk for chronic lifestyle diseases later on. It is important to determine the weight for age in children under five years of age, as they are most vulnerable to all forms of malnutrition including stunting [47].

Globally stunting affects 1 in 4 children under five years and 40% of children in SSA [46]. According to the NFCS-FB-1 study (2005), 1 in 5 children in South Africa were stunted. According to the NFCS-FB-1 study, children living in rural areas were most affected by stunting [27]. Moreover, the prevalence of stunting was, respectively, 24% and 26% in the UMkhanyakude and Zululand districts of KZN [48]. These results are similar to the results reported in a more recent study, the SANHANES-1 study [20]. Between 2005 and 2012, stunting in South African children aged 1–3 years has worsened as the rates have increased from 23.4% to 26.5% [14,20]. There are many implications of stunting, especially in children under 5 years of age.

Stunting can lead to poor cognitive, motor and social-emotional development [49,50]. From foetal development up to two years of age, children are at risk of poor development due to inadequate nutrition. After two years, some of these effects become irreversible [51]. These children tend to perform poorly in school, suffer from food and nutrition insecurity as well as lack financial stability. This contributes to an economic loss as these children grow up to become adults that are unemployed and rely on social grants, thus a viscous cycle of poverty and food insecurity continues may be perpetuated [52].

Based on the available literature for South Africa, it is evident that stunting is a serious problem in South Africa and KZN in particular. It would thus be beneficial to determine the nutritional status of people living in KZN. This information would assist to improve the nutritional status in children under 5 years and could contribute to breaking the poverty cycle. Poverty remains a major determinant for poor nutritional status as it limits access to nutrient dense and diverse foods. Adapting agricultural as a vehicle for addressing food and nutrition security remains the most viable option. As mentioned earlier, food gardens and communal gardens could be means of obtaining nutrient rich foods that would help improve the stunting situation in South Africa and KZN. However, agriculture should only be viewed as part of a solution not the panacea. It needs to be supported by other transdisciplinary interventions.

##### Underweight and Wasting

Underweight and wasting are defined as a weight for age and a weight for height less than the minus 2 SD, respectively. Both underweight and wasting are serious nutritional problems that may be caused by either weight loss or poor nutritional intake [42]. Globally, 16% of children under five years are underweight; the figure is higher in SSA with 21% of children under five years being underweight [44]. Although these percentages are high, an improvement in the percentage of underweight and wasting has been observed in South Africa. National studies conducted for the period 2005–2012 indicated that the prevalence of underweight and wasting in children aged 1–3 years in South Africa declined from 11% to 6.1% and 5.1% to 2.2%, respectively [14,20]. In KZN, the highest incidence of underweight children between the ages of 12–23 months was found in the UMkhanyakude district (6%) [29].

As alluded to earlier, poor dietary intake may be a contributing factor to malnutrition. The improvement in undernutrition in KZN could be attributed to the Integrated Management of Acute Malnutrition (IMAM) programme that was implemented by the national Department of Health in 2014 [53]. Even with the improvement in the number of people that are underweight and wasted, it remains important to determine the nutritional status of vulnerable children and adults in KZN using anthropometric measurements, especially as rural people live far away from institutions that provide basic health care services. Many of malnourished poor rural people may remain undetected, as they may have not visited a health care facility [50]. Further, Protein Energy Malnutrition (PEM), a common form of malnutrition is not well-documented. Literature on its prevalence in South Africa and KZN remains scanty. There are limited studies and/or specific statistics that focus on PEM on its own. Studies usually focus on wasting, stunting and undernutrition and link these back to PEM. Thus, there is a gap in the literature with the reporting of these statistics. It is important to determine the nutritional status of the rural KZN population and possibly identify those with PEM using anthropometric measurements. This would assist in improving the nutritional status of those with malnutrition.

##### Micronutrient Deficiency—Vitamin A Deficiency (VAD)

Micronutrient deficiencies are also a problem in developing countries but are not routinely treated. Globally, about two billion people have micronutrient deficiency due to the consumption of poor quality foods that lack diversity. The most common micronutrient deficiencies experienced in developing countries are iron, iodine, zinc and vitamin A [6,17]. Micronutrient deficiencies result in several health conditions, including growth retardation and delayed development [6]. Labadarios [14] showed that children in South Africa were deficient in the following micronutrients: vitamin A, calcium, iron, zinc, folate, vitamin B6, niacin, riboflavin, vitamin C and vitamin E. The study further indicated that, in South Africa, the highest prevalence of micronutrient deficiencies was in rural communities [53]. Similarly, the NFCS-FB-1 2005 study also found that many micronutrients were consumed at less than 67% of the recommended dietary allowances (RDA) [14]. These also included calcium, iron, zinc, selenium, vitamin D, vitamin C, vitamin E, riboflavin, niacin, folic acid and vitamin D [27]. Nationally, 43.3% of children in South Africa have a poor zinc status [27].

Iron: On a national level, there was a poor iron status in 1 in 5 and 1 in 10 women and children, respectively. Iron deficiency anaemia affected 5.9% of children aged 1–9 years in KZN [27]. Iron deficiency has improved since the SAVACG study conducted in 1994 as the number of persons with iron deficiency decreased from 5% to 1.9% [18,19,20]. A recent longitudinal study conducted on children aged 4–6 years and 6–8 years in rural KZN found that out of the 103 children in the study, 37.9% of the children were mildly anaemic, 60.2% were moderately anaemic and 1.9% were severely anaemic [54]. These study results were in contrast to the results obtained from the 2012 SANHANES-1 study. A possible reason for the difference with results could be due to the classification used to analyse data. The study conducted by Gwetu et al. [54] classified anaemia into two target groups 0–59 months (mild: 10–10.9 g/dL; moderate: 7–9.9 g/dL; and severe: <7 g/dL) and 5–11 years (mild: 11–11.4 g/dL; moderate: 8–10.9 g/dL; and severe: <8 g/dL) [54]. The 2012 SANHANES-1 study classified children under 5 years (mild: 10–10.9 g/dL; moderate: 7–9.9 g/dL; and severe: <7 g/dL) [20] Gwetu et al. [54] reported that the results of their study were similar to previous national studies such as the SAVAGC and NFCS-FB-1) [18,27], and local studies [55,56]. These all indicated that the prevalence of anaemia ranged from 16.5% to 33% [18,27,55,56]. As previously highlighted, the SANHANES-1 study used disproportionate stratification that did not cover every area of a specific province. This may explain, in part, the differences noted.

A separate study that was conducted in the urban population in Durban, KZN also found high rates of anaemia. The cross-sectional prospective study that was conducted in a regional hospital found that out of 2000 pregnant patients enrolled in the study, 854 patients (42.7%) were anaemic. From the pregnant women that were anaemic, 81.4% were mildly anaemic, 18% were moderately anaemic and 0.6% were severely anaemic [57]. Anaemia in pregnancy is a significant problem as it affects both the mother and unborn child’s health. Iron is required during pregnancy to assist with foetal growth and improves Apgar scores. A mother suffering from anaemia is at risk of maternal and perinatal mortality due to poor nutritional status [58,59]. It can result in a low birth weight premature infant [58]. This is a serious problem as the vicious cycle of malnutrition will continue. These infants may suffer from development delays that will affect their performance in school. This could result in unemployment later in their lives hence exposing them to poverty and food insecurity.

These results indicate that although an improvement in the iron status was observed at a national level, it is important to highlight that national studies only sample a portion of the province and they are not district specific. It also indicates that iron deficiency anaemia affects two vulnerable groups in KZN, women of childbearing age and children. The nutritional status of these two vulnerable groups should be prioritised in KZN as they are at a high risk of micronutrient malnutrition. A possible long-term strategy that could be explored to help address micronutrient malnutrition is dietary diversification through promoting household and community food gardens, utilisation of wild vegetables and biofortification of commonly consumed foods.

Vitamin A: Vitamin A is a common micronutrient deficiency observed globally and throughout SSA. A few national studies have documented the state of vitamin A deficiency (VAD) in South Africa. In 1994, the SAVACG study Labadarios [18] reported that 33.3% of South African children had VAD while the NFCS-FB-1 study Labadarios [27] conducted in 2005 reported that 63.6% had VAD. This showed that the VAD situation had worsened between 1994 and 2005. Furthermore, the NFCS-FB-1 study reported that two in three women had VAD. In KZN alone, six out of ten women were vitamin A deficient, which was the highest in South Africa. Forty-four percent of children between the ages of 1–9 years living in KZN were VAD. Overall, KZN had the second highest prevalence of VAD [18,19,27]. A more recent study, the SANHANES-1 study Shisana et al. [20] conducted in 2012, reported an improvement in the VAD situation in South Africa. Although still significantly high, the SANHANES-1 study reported a decline in the number of children with VAD (43.6%). This study also reported that 45.5% of rural South Africans were VAD, which was the highest in the country. The SAHANES-1 study also reported that there was an improvement in the vitamin A status of females residing in KZN. Persons with the lowest education levels were the most affected by VAD [20].

Although there have been improvements in the number of people with VAD, it remains a challenge in South Africa, especially KZN. It is important to note that VAD could affect protein synthesis, vision, growth and development [60,61]. VAD results in children not been able to reach their full potential as well as repetition of the vicious cycle of malnutrition. Yet again, it is evident that poor socio-economic status and limited access to nutritious foods results in micronutrient deficiencies such as VAD. Diversifying household food baskets through exploiting underutilised crops could assist. However, the acceptability of such crops by poor rural communities remains an obstacle. It is therefore important to identify the foods consumed by people at risk of VAD in KZN and analyse these foods to obtain the nutritional adequacy.

The improvements in the percentage of micronutrient deficiencies noted could be attributed to the success of the nutritional interventions in place to help alleviate micronutrient deficiencies. Although these interventions have shown an improvement in the percentages of people affected by micronutrient deficiencies, a slow progression has been observed. Despite the improvement seen in the national studies with the iron status in women and children, it is evident that it remains a problem in some areas of KZN. With the high poverty rates and the worsening economy, many poor rural communities remain unable to access basic necessities for living. This leads to poor nutritional status as foods consumed lack variety and many essential nutrients. By assessing the dietary intake of the rural population in KZN, one could improve their dietary intake by modifying current eating patterns or assisting with cheaper alternative options. Recipes of current diets could be documented and assessed for nutritional composition. Items from these recipes could be substituted with options that are more nutritious. For example, substituting unfortified white maize with yellow maize or sweet potato with orange-fleshed sweet potato could address VAD. Adding fruits and vegetables to diets that lack variety by promoting vegetable gardens, utilising wild vegetables or biofortified foods could assist in diversifying household food baskets.

#### 3.1.2. Over-Nutrition

For many years, undernutrition has been under the spotlight while over-nutrition received less attention. However, this has now changed [52]. Over-nutrition was previously mostly associated with affluence. However, due to the nutritional transition, it is now also prevalent in middle and low-income groups. Over-nutrition is a major risk factor for chronic lifestyle diseases, especially in women [62].

##### Overweight and Obesity

Children: There are several possible causes for childhood obesity. These include increased consumption of poor quality high-energy foods, children born to mothers who were overweight or obese during pregnancy, overweight or obese parents, poor physical activity and metabolic disorders [40]. Overweight and obesity are a serious health concern, especially in children, as it increases risk of other chronic lifestyle diseases later on such as cardiovascular diseases, hypertension, and diabetes mellitus [40].

The Labadarios et al. [14] found that 1 in 10 South African children were overweight. A separate study conducted by Armstrong et al. [63] using random sampling in South African children aged 6–13 years in five selected provinces during the national health survey (2001–2004), found that the prevalence of overweight and obesity increased with age in black girls whereas the opposite trend was observed with white girls. For the black girls, the combined percentage of overweight and obesity increased from 11.9% in black girls aged 6% to 21.8% in black girls aged 13 [63]. Similarly, a national study conducted in 2012 in South Africa reported that the prevalence of overweight and obesity was lower in boys (16.5% and 7.1%, respectively), than girls (16.5% and 4.7%, respectively). These results were consistent for all age groups. KwaZulu-Natal was one of the provinces with high rates of obesity. The prevalence of obesity in KZN was 6.1% and 8.5% in girls and boys, respectively. Furthermore, this study reported that black girls were the most affected by obesity [20].

Adults: Black African women are the ethnic and gender group that was reported to be at greatest risk of obesity in South Africa [64,65]. Another study that reviewed demographic and health surveys to assess the nutritional status in seven African countries found that in Africa, overweight and obesity were on the rise [66]. Poor women and those that had less than a primary school education were the most affected by overweight and obesity [66]. A similar trend was observed in South Africa. The SANHANES-1 study reported that, on a national level, the prevalence of obesity in women from the rural formal and rural informal populations was 31.8% and 37.6%, respectively. Furthermore, KZN was shown to have the second highest prevalence of obesity. The prevalence of overweight and obesity was higher in women (24.8% and 39.2%, respectively), than men (20.1% and 10.6%, respectively) [20].

There have been a few studies conducted to assess the nutritional status in the KZN population (Table 3). Limited data has been collected from the urban KZN population. Many authors reported that stunting affected young children whereas overweight and obesity were a major problem, especially among females from both urban and rural areas of KZN (Table 3). It was noted that the prevalence of overweight and obesity increased with age in black females [41,67,68,69,70]. As highlighted earlier, the rates of stunting in children age 1–3 years have worsened from 2005–2012 from 23.4% to 26.5% (Table 3). A study conducted by Grobbelaar et al. [70] reported that children consumed foods that were high in energy and carbohydrates and inadequate in micronutrients (vitamin C, calcium and iodine) which are essential for growth and development. Poor dietary diversity may be a possible reason for the high rates of chronic undernutrition in the form of stunting.

The studies reviewed indicated that females were more affected by overweight and obesity than males. There are many possible reasons for the increasing prevalence of obesity in older girls of black ethnicity. Some studies have noted that overweight is perceived as being associated with wealth and a negative HIV status [71]. Thinness was usually associated with illness and being HIV positive [65,68]. From the studies reviewed, it was noted that some overweight and obese participants did not perceive themselves as overweight or obese. Furthermore, the participants did not recognise that they had a weight problem [68,71]. These social misconceptions further emphasise the need for education and awareness programs, especially in poor rural communities, to empower people to make informed dietary decisions. Such programs should also provide healthy solutions, which are available and accessible to people living in rural areas. Over-nutrition predisposes people to a high risk of chronic lifestyle diseases [71].

Dietary reasons for both over and under-nutrition: Various studies conducted in KZN have noted that although participants consumed high portions of foods, these foods were nutritionally inadequate. These foods were often high in energy, carbohydrates and sodium but low in micronutrients such as vitamin A, B12, C, D, E, zinc, pantothenic acid, calcium and iodine. There was a poor intake of fruits, vegetables and dairy products. Studies have also noted a poor intake of dietary fibre [70,72,73]. These results indicate that dietary diversity is a problem. Part of the solution may be to identify sources of food in rural areas and to broaden this base. In this regard, food gardens at both a household level and community level have the ability to bridge the gaps in nutrition and contribute to improving nutritional status of vulnerable groups.

Modi et al. [26] reported that there were several indigenous crops in KZN; these could be utilised as food sources to diversify diets. Utilisation of these crops could provide nutrients that are currently lacking in diets of poor rural people. For example, amaranth, black jack and gallant soldier are popular indigenous crops in the Ezigeni area of KZN. Of these, amaranth contains vitamin C while black jack contains vitamin A and E (Modi et al., 2006). As such, they could be used to diversify diets, especially where other vegetables were not found abundantly [26]. Mabhaudhi et al. [74] further emphasised that improved utilisation of underutilised crops could go some way in improving dietary diversity and providing nutrient dense foods in poor rural communities.

Based on the literature reviewed, it is evident that there is limited data on the nutritional intake of people living in KZN. It is also important to highlight that these studies were conducted in specific districts and sub-districts within KZN and therefore do not necessarily reflect the nutritional status of the KZN population as a whole. Most of the localised areas within KZN have not been investigated. Each area has a population with a different income, socio-economic status and education level. These factors could possibly affect the nutritional status of the populations living within these districts. Therefore, it would be beneficial to investigate the nutritional status of people living in the districts and sub-districts that have not been previously investigated.

### 3.2. Nutritional Interventions in KwaZulu-Natal

In response to the nutritional challenges, there have been a number of interventions employed by the South African government in order to combat nutritional deficiencies. These include food fortification, vitamin A supplementation, the IMAM outpatient supplementation and dietary diversity [21,27,77,78].

#### 3.2.1. Food Fortification

The results of the NFCS study in 1999 led to the fortification of foods to assist in improving the nutritional status of South Africans [19]. Food fortification is a cost-effective process that involves the addition of micronutrients to foods that are commonly consumed by vulnerable populations [79,80]. As of October 2003, the South African Department of Health made it mandatory for all maize and wheat flour to be fortified with vitamin A, iron, zinc, folic acid, thiamine, niacin, vitamin B6 and riboflavin [78]. This decision was based on the fact that the commonly consumed foods in South Africa were bread and maize [19]. However, access to fortified foods remains problematic for poor people who rely on social grants to purchase food [60,81]. This implies that alternative solutions are needed to reach this vulnerable group.

Although earlier statistics have indicated an improvement in various forms of malnutrition, it is still questionable whether this long-term strategy has improved nutritional status. It would be beneficial to identify the types of fortified and unfortified foods consumed by the rural people in KZN in order to obtain information on their nutritional intake. This data is important as foods are now fortified since October 2013. If these people are unable to utilise fortified foods, they will consume unfortified foods that lack adequate micronutrients. Biofortification is a complementary strategy that involves the production of micronutrient crops [82,83]. This strategy could be introduced to assist in improving the nutritional intake of poor rural people. However, before this is implemented, there is a need to determine the acceptability of biofortified foods in KZN. Current studies indicate that there is low acceptance of biofortified foods [37,84,85]. However, there have also been reports of success. Govender et al. reported that biofortified maize was acceptable to mothers if it had a health benefit and was affordable and readily available [86]. Thus, there is a possibility that biofortified foods could be accepted in certain areas of KZN.

#### 3.2.2. Vitamin A Supplementation

Vitamin A supplementation was another strategy employed by the South African Department of Health to assist in the alleviation of VAD. The KZN DoH guidelines state that all children over six months of age and under five months should receive a routine dose of vitamin A supplementation [77]. A therapeutic dose is issued to all children that present to the hospital or clinic with severe malnutrition or signs of VAD [77]. Although this process has been implemented and is currently in place in KZN, not all children benefit from it. Healthcare facilities are usually far from where people reside and many people do not have money to transport children to healthcare facilities for supplements. Many children only receive a therapeutic dose of vitamin A on admission to hospital with malnutrition [77]. Results of a study conducted in KZN indicated that there was poor utilisation of health care facilities. A high percentage of mothers (25%) living in KZN had no access to basic healthcare and in these areas many home deliveries were noted [39]. Another study conducted in UMkhanyakude and Zululand indicated that not all the children residing in these areas received vitamin A supplementation [29].

As alluded to earlier, other than financial constraints, poor maternal education is also a contributing factor to poor nutritional intake. Poor utilisation of health care facilities to obtain vitamin A supplementation may be attributed to low levels of education. Caregivers from these communities may not be aware of the importance of vitamin A supplementation [87], thus resulting in poor nutritional status of children in these areas. Maternal education could play a significant role in reducing malnutrition, as mothers could be educated on utilisation of basic resources effectively and family planning. This will result in a reduction of overpopulation and improved nutritional status in children [6]. There seems to be a gap in determining the role of nutritional education in the improvement in the nutritional status of the KZN rural population; this aspect could be further explored.

#### 3.2.3. Integrated Management of Acute Malnutrition Outpatient Supplementation

Protein energy malnutrition is a concern in KZN. In most cases, there is poor utilisation of health care facilities due to location as well as financial constraints. Many caregivers only bring their children to healthcare facilities when they are ill and, in most cases, the children are severely malnourished. On discharge from hospital, all malnourished children (severe acute malnutrition, moderate acute malnutrition and not acutely malnourished but at risk, receive therapeutic supplementation to take home in order to assist with the recovery process. Caregivers are educated appropriately and follow-up appointments with dietitians, nutritionists or nutrition advisors are done. This supplementation programme also caters for undernourished adults as well as pregnant and lactating women. Different supplements are issued depending on the severity of the undernutrition and the age of the individual [51]. This program could be a contributing factor in the improvement in wasting and underweight observed in KZN.

### 3.3. Dietary Diversity

Dietary diversity is a long-term strategy used to assist in combating micronutrient deficiencies in South Africa [88]. Dietary diversity involves adding a variety of foods to the diet such as fruit and vegetables, legumes, starch and animal products [89]. In South Africa, KZN is the province with the highest energy, protein, fat carbohydrates and fibre intake, however, micronutrient intake is poor [19]. Labadarios [14] found that one in two children consumed half of the RDA for micronutrients; calcium, iron, zinc, selenium, vitamins A, D, C and E, riboflavin, niacin, vitamin B6 and folic acid. Unfortunately, the majority of people living in tribal and informal urban areas in South Africa, specifically KZN, have a diet that lacks dietary diversity. They consume foods that lack nutrients and are deficient in energy.

The commonly consumed foods in South Africa are mealie meal, white sugar, tea, brown bread, non-dairy creamer, brick margarine, chicken meat, full cream milk and dark green leafy vegetables [19,53]. A study conducted in a peri-urban site in Marianhill, Pinetown by Faber et al. [90] showed that commonly consumed foods were sugar, maize meal porridge, bread, rice, cordial squash, hard margarine, tea and legumes, similar to other studies. On the other hand, the foods that were consumed by more than half the participants in rural KZN were maize meal and bread [48]. From this it is evident that many of these diets are low in eggs, legumes, animal products and vitamin A-rich fruit and vegetables, due to the high costs of these foods.

## 4. Linking Agriculture to Addressing Malnutrition

The traditional crops grown in KZN are maize, beans, potatoes, pumpkins, taro and groundnuts [26]. This traditional food basket highlights a lack of dietary diversity. Due to the high costs of food, household and community food gardens are now being promoted as an alternative means of improving availability and access of nutritious foods in poor rural households. The study conducted by Modi et al. [26] found that wild vegetables contributed to the nutritional intake of the participants from Ezigeni, a rural location in KZN. Wild vegetables are found in abundance when other vegetables are scarce [26]. KwaZulu-Natal is the province with the second highest consumption of wild vegetables with as many as 24 species of wild vegetables being identified in KZN. The authors noted that although these vegetables were available, there has been a decline in their consumption. This could be attributed to the nutrition transition from traditional diets to more westernised diets. If wild vegetables were consumed, this could improve food insecurity by providing variety to a diet that is already nutrient deficient [26,91].

Although wild vegetables are not routinely consumed and communal gardens are not popular, many people grow foods for their own consumption. A study conducted by Faber et al. [92] found that cabbage and pumpkin were popular consumed items. Fifty percent of the participants consumed cabbage and 63% grew their own pumpkin [90]. Yellow vegetables were only consumed by a small percentage of the children in KZN, which could be the reason for the poor vitamin A status [48]. A possible intervention to assist in improving the nutritional status of vulnerable groups is to utilise wild vegetables. There has been a decline in the consumption of these affordable and often free crops due to lack of education. These crops are more often nutrient dense and readily available. There are, however, challenges facing the acceptability of some of these indigenous leafy vegetables. The acceptability of indigenous crops could be improved if people residing in rural communities were made aware of the health benefits of utilising these crops and incorporating them into already existing meals. There is not much literature on the effect of education on the acceptability of wild vegetables and other indigenous crops but this could possibly be explored.

There are a number of factors that limit crop production in poor rural communities of which water is the major limiting factor. Adequate, clean and safe water is vital for agricultural production, food processing and preparation. Access to clean and safe water remains a challenge issue in rural KwaZulu-Natal and this directly affects crop yield [27,93]. Rural communities use different water sources for agricultural production and food processing and preparation. As such the adequacy, quality and safety of the water likely varies. The link between food production and water is an important one as it directly effects food and nutrition security [94]. In this regard, the use of indices such as nutrition water productivity (NWP) could be useful for identifying crops that offer the most “nutrition per drop of water”. It would be beneficial to investigate the link between water, agriculture and nutrition in KZN and the subsequent utilisation of a diverse set of nutrient dense crops to improve dietary diversity especially during times of water scarcity.

## 5. The Way Forward

Despite the nutrition interventions, more work is required in order to improve the nutritional status of poor rural communities in KZN. The Sustainable Development Goals (SDGs) 1, 2 and 3 speak explicitly to the need to (i) end poverty; (ii) achieve zero hunger; and (iii) good health and well-being [95]. Achieving these goals is important and discussions on how to do that currently dominate the post-2015 debate [69,94]. It is evident that food and nutrition security remains a major problem. A multi-disciplinary approach needs to be adapted to help in addressing poor nutrition. Agriculture on its own is insufficient to provide nutritious produce. If there is not proper utilisation of nutritious foods, there will be no change in the nutritional status of vulnerable people.

Malnutrition is a serious health concern, especially in children. A major contributor to poor nutritional status is poverty. Financial insecurity directly affects the utilisation of foods, which affects the nutritional status of vulnerable groups [96]. The Malabo Declaration [97] noted that agriculture should be recognised as provider of nutrition and not as a provider of food. This emphasises the crucial role that agriculture and proper utilisation plays in improving the nutritional status of vulnerable populations. Before one can determine what proper utilisation is for a specific population, one needs to investigate what foods are consumed within that population group. Thus, it is important to determine the food intake and sources of food of poor rural communities in KZN and use this as a foundation to strengthen and diversify their food systems.

While the current review could have benefitted from discussion about the reasons behind the prevalence of both over-nutrition and undernutrition in KZN- including discussing the potential role of “catch up growth” in stunted infants, information on stunting remains scant in South Africa. This is because stunting is not routinely treated in South African government hospitals. The current malnutrition programme focuses on wasting (weight for height and mid upper arm circumference) which links to PEM where there is limited data available. As a way forward, data on stunting should be collected and recorded throughout government hospitals. This would also contribute to monitoring of progress related to the implantation of the SDGs.

## 6. Conclusions

The trends of the nutritional status of population groups in the province of KZN are similar to national trends. There is a burden of both under- and over-nutrition. Undernutrition, specifically stunting, remains a problem in children. Over-nutrition, both overweight and obesity, are a problem among black African females. Data is limited on the micronutrient status of both the rural and urban population. Data on the nutritional status of the different population groups in the KZN are also very limited. There was a gap in information relating to the nutritional status of rural population groups in the local municipalities of uMshwathi, uMsinga and eThekwini, in uMgungundlovu and uMzinyathi Districts and the eThekwini metropolitan of KZN that are the focus of the current study. This was attributed to the fact that national studies aimed to determine the nutritional status of the South African population and not specific sub-groups within the population. Consequently, while such studies are plausible, they have limitations of over generalizing the nutritional status specific sub-groups.

Availability and access to nutritious foods is a major obstacle to improving diets of poor rural people. The majority of them rely on social grants which restrict them to a narrow food basket that lacks diversity. Under these circumstances, agriculture offers an opportunity to address the availability and accessibility of diverse nutrient dense foods in poor rural communities. The promotion of household and community food gardens should be encouraged. However, access to water remains a limitation to crop production, especially in rural areas. Thus, agricultural interventions in these areas should also be water smart. In that regard, the inclusion of underutilised indigenous crops, such as indigenous leafy vegetables, offers opportunities to improve the nutritional water productivity of rural cropping systems. The link between, water, food, nutrition and health should be strengthened through multidisciplinary interventions.

## Figures and Tables

**Table 1:** Districts of KwaZulu-Natal province and their local municipalities ([21,22]).

District	Local Municipalities
Ugu	Ezinqoleni, Hibiscus Coast, uMdoni, uMuziwabantu, uMzumbe and Vulamehlo
uMgungundlovu	Impendle, Mkhambathini, Mpofana, Msunduzi, Richmond, uMngeni and uMshwathi
uThukela	Emnambiti/Ladysmith, Imbabazane, Okhahlamba and uMtshezi
uMzinyathi	Endumenil, uMsinga, Nquthu and uMvoti
Amajuba	Dannhauser, eMadlangeni and Newcastle
Zululand	AbaQulusi, eDumbl, Ulundi and uPhongolo
Umkhanyakude	Hlabisa, Jozini, Mtubatuba, The big 5 False Bay and uMhlabuyalingana
UThungulu	City of uMhlathuze, Mthonjaneni, Nkandla, Ntambanana, uMfolozi and uMlalazi
ILembe	KwaDukuza, Mandeni, Maphumulo and Ndwedwe
Harry Gwala (previously known as Sisonke)	Greater Kokstad, Ingwe, kwaSani, Ubuhlebezwe and uMzimkulu

**Table 2:** Classification of malnutrition using anthropometric indicators [43,44].

Age	Classification	Indicator
Under 5 years (MUAC not done in under 6 months)	SAM	WFH < −3 SD; MUAC < 11.5 cm
	MAM	WFH < −2,−3 SD; MUAC 11.5–12.4 cm
	NAM at risk	WFH < −2 SD; MUAC > 12.4
5–9 years	SAM	BMI < −3 SD MUAC < 13.5 cm
	MAM	BMI for age ≥ −3 SD– < −2 SD MUAC 13.5–14.5 cm
10–14 years	SAM	BMI < −3 SD MUAC < 16 cm
	MAM	BMI for age ≥ −3 SD– < −2 SD MUAC 16–18 cm
>15 years	SAM	BMI < 16 kg·m^2^ MUAC < 21 cm
	MAM	BMI 16–18.5 kg·m^2^ MUAC 21–23 cm
Pregnant and lactating woman	SAM	MUAC < 21 cm
	MAM	MUAC 21–23 cm

SAM = Severe Acute Malnutrition; MAM = Moderate Acute Malnutrition; WFH = Weight for Height; NAM = Not Acutely Malnourished; BMI = Body Mass Index; MUAC = Mid Upper Arm Circumference; SD = Standard Deviation.

**Table 3:** Studies conducted to assess the nutritional status of the KZN population.

Authors	Study Design and Methods	Area Conducted	Participants	Findings
Napier and Oldewage-Theron [69]	Three informal settlements were randomly selected. Anthropometric data was collected (weight and height) and a structured 24-h recall was conducted.	eThekwini municipal district (Urban area)	Girls in secondary school and women aged 19–28 years of age (*n* = 523)	Stunting was evident in young girls (7.7%). Forty-three percent of the girls were at risk of being overweight and 12.8% were overweight. BMI for age indicated that 5.2% of the women were underweight and that 30.5% and 15% were overweight and obese respectively. Half the women had a normal BMI. The intake of micronutrients was adequate in both the girls and women, however, the energy intakes were inadequate.
Duncan et al. [68]	Nested cross sectional study. Anthropometric measurements and blood pressure were measured for all participants. A questionnaire was formulated and participants interviewed.	Manguzi, KwaZulu-Natal (Mahlungulu, Maputa, Mshundu, Thengane and Zama Zama)	109 males and 391 females. Patients from 11 primary healthcare clinics	The results of the study indicated that 28% of the participants were overweight, 34% were obese and 4% were underweight. This study concluded that most of the participants were overweight and obese; however, not many participants perceived that they were overweight.
Devanathan et al. [71]	Cross sectional exploratory study. Systematic sampling was used. Anthropometric measurements were taken and an interview was conducted.	Wentworth Hospital, Durban, KZN	328 urban black women aged 19–70 years	The prevalence of overweight and obesity was 16% and 76% respectively. All participants had one or more chronic diseases of lifestyle. The overweight and obese women who had one or more chronic diseases of lifestyle perceived themselves as thinner than they were.
Grobbelaar et al. [70]	Anthropometric measurements were conducted. Seven-day cycle menu was obtained and analysed.	Three residential care facilities Durban	33 girls and 110 boys aged 5–18 years	Severe stunting was noted in 4.7% and 3.3% of the boys aged 4–8 years and 14–18 years, respectively. Stunting affected 13.3% and 20% of girls aged 9–13 years and 14–18 years, respectively. Wasting was noted in 6.7% of girls aged 9–13 years and 3.3% of boys aged 14–18 years. 26.7% of girls aged 14–18 years were overweight and 33.45% of girls aged 9–13 years were at risk of becoming overweight. This study found that younger boys were more overweight than younger girls were and the opposite was noted for older boys compared to girls. These authors found that majority of the children consumed all the foods on their plate. The energy, protein and carbohydrate intakes met 100 percent or more of the Dietary Reference Intake (DRI). Calcium and iodine requirements were not met by the children. Further, low intake of vitamin C was noted in both the older girls and boys. Recommended fibre intakes were not met by any of the groups. Results from this study showed that fruit and vegetable intake was limited. On average, a single serving of 40 g of vegetable was given to the children whereas fruit was only given three times a week. This study concluded that although large portions were given to the children the foods were nutritionally inadequate and there was poor intake of fruits, vegetables, milk and milk products.
Kolahdooz et al. [73]	Cross-sectional study assessing dietary adequacy from a 24-h recall. Participants were randomly selected.	Empangeni, KZN	136 rural adults (52 males and 84 females)	Energy content of both male and female diets exceeded the DRI (2200 and 1800 kcal, respectively). Mean daily energy intake from carbohydrate intake of both males and females was higher than the DRI (69% and 66%, respectively). This study indicated that although the protein intake was adequate, plant sources of protein were consumed by the majority of the subjects. This study showed that the male participants consumed inadequate amounts of vitamin A, B12, calcium and zinc. The sodium intakes in all groups were higher than the DRI. This study concluded that despite food fortification in South Africa, the majority of the study population consumed diets that contained inadequate amounts of vitamin A, B12, C, D and E, calcium, zinc and pantothenic acid.
Tathiah et al. [75]	Secondary analysis of anthropometric data (weight and height) collected during the HPVVDP in Zululand, SA during 2011.	Nongoma and Ceza, Zululand	Girls aged 9–14 years	There was a high prevalence of stunting in the age group 11–12 years. More than 50% of children aged 13–14 years were stunted. Overall, 9% were overweight, 3.8% were obese, 4% were underweight and 9.2% were stunted. Both under and over nutrition was noted in girls between 9–14 years residing in two rural areas of KZN.
Spearing et al. [72]	Random selection of persons living in rondavels of the same socioeconomic status. Data obtained for the recipes were analysed by Nutribase clinical Nutrition Manager, version 9.	Rural village surrounding Empangeni, KZN	34 males and 45 females that prepared or purchased foods	Commonly consumed composite dishes were; fried beef, beef stew, beef soup, fried chicken, chicken soup, chicken stew, fish stew, dumplings, jeqe, phutu, potatoes, stiff pap, beans, samp and beans, fried spinach and fried cabbage. The study found that participants’ diets contained good sources of protein, vitamins and minerals; however, it was high in fat.
Zhou et al. [76]	A large population based survey measuring BMI and blood pressure.	Hlabisa sub-district in rural UMkhanyakude	BMI (2298 participants) and Blood pressure (2307 participants) Females aged 15–49 and males aged 15–54	More than half of the participants were overweight (58.4%). This study showed that females were more likely to be overweight in comparison to their male counterparts.
Schoeman et al. [29]	A cross-sectional study was conducted. Structured interview questionnaires were used and anthropometric measurements were taken (height and weight).	Umkhanyakude (*n* = 398) (sub-district Jozini), Zululand (*n* = 303) (sub-district Pongola) and Oliver Reginald Tambo (OR) Municipality (*n* = 364) (sub-district Nyandeni)	Children between 0–59 months fromUMkhanyakude, Zululand and OR Tambo	Thirty percent of participants in the two KZN districts had food gardens. Half of the participants from the two KZN districts had experienced a food shortage in the previous 12 months. Zululand had the lowest coverage of vitamin A supplementation. Wasting was not a concern in this study. The highest rates of stunting were seen in UMkhanyakude (6%) in the 12–23-month old group. Stunting was higher in the second year of life. The rates of overweight in 0–23-month group was higher than underweight. There was a high rate of obesity noted among the caregivers in the study (UMkhanyakude = 42%, Zululand = 60%).
Smuts et al. [39]	A cross-sectional study was conducted. A questionnaire was used and anthropometric measurements were taken.	OR Tambo and Alfred Nzo district (Eastern Cape, *n* = 1794) and UMkhanyakude and Zululand (*n* = 1988)	Children 0–71 months old and caregivers	Between sixteen and eighteen percent of the children in both provinces were overweight. Childhood malnutrition was seen to double from the first year of life to the second. Further, the prevalence of stunting was significantly high in the Nongoma district of KZN. The mean BMI for the caregivers were above 25 kg/m^2^ for all areas except the UMkhanyakude district. Obesity was higher among females; 45% of female caregivers in KZN were obese. Only 9% of the caregivers in the UMkhanyakude district were underweight. This study indicates that maternal over nutrition and childhood malnutrition co- exist in both the Eastern Cape and KZN.
